# Cardiovascular safety for once-weekly dulaglutide in type 2 diabetes: a pre-specified meta-analysis of prospectively adjudicated cardiovascular events

**DOI:** 10.1186/s12933-016-0355-z

**Published:** 2016-02-24

**Authors:** Keith C. Ferdinand, Fady T. Botros, Charles M. Atisso, Philip T. Sager

**Affiliations:** Tulane University SOM, 1430 Tulane Avenue, #8548, New Orleans, LA 70112 USA; Eli Lilly and Company, Indianapolis, IN 46285 USA; Cardiac Safety Research Consortium, Stanford University School of Medicine, 719 Carolina St., San Francisco, CA 94107 USA

**Keywords:** Type 2 diabetes, Meta-analysis, Glucagon-like peptide-1 (GLP-1), Incretin, Cardiovascular events, MACE

## Abstract

**Background:**

Patients with type 2 diabetes (T2D) have a substantial increased risk for cardiovascular (CV) disease and associated mortality than those without diabetes. Dulaglutide is a once-weekly glucagon-like peptide-1 receptor agonist that is approved for treatment of T2D.

**Methods:**

This meta-analysis evaluates the CV risk in patients with T2D treated with dulaglutide in 9 randomized safety and efficacy trials. Mean (median) treatment duration was 333 (358) days. Reported CV events were independently adjudicated by a treatment-blinded clinical endpoint committee. The primary measure was a 4-component major adverse CV event (4-component MACE) composite endpoint of death due to CV causes, nonfatal myocardial infarction (MI), nonfatal stroke, or hospitalization for unstable angina. Additional pre-specified endpoints included adjudicated coronary revascularizations, hospitalization for heart failure, and all-cause mortality. A Cox proportional hazards regression model (stratified by study) was used to estimate the hazard ratio (HR) and confidence interval (CI). Tests of treatment effects for the primary endpoint were conducted at a 2-sided alpha level of 0.0198 and a corresponding 98.02 % CI was calculated. Statistical heterogeneity between the strata (studies) was tested by including in the Cox model an interaction term between treatment and strata.

**Results:**

The analysis included 6010 randomized patients [dulaglutide: 3885; comparator therapy (active or placebo): 2125]; cumulative exposure to dulaglutide or comparator therapy was 3941 and 2223 patient-years, respectively. The demographic and baseline CV disease characteristics were similar across groups. Twenty-six (0.67 %) patients in the dulaglutide group versus 25 (1.18 %) in the comparator group experienced a primary 4-component MACE (HR 0.57; adjusted 98.02 % CI 0.30, 1.10). Results for the 3-component MACE (composite endpoint of death due to CV causes, nonfatal MI or stroke), 6-component MACE (composite endpoint of death due to CV causes, nonfatal MI or stroke, hospitalization for unstable angina or heart failure, or coronary revascularizations) and all-cause mortality were consistent with the primary analysis (HR < 1.0 for all).

**Conclusions:**

These results suggest that dulaglutide does not increase the risk of major CV events in T2D patients. The ongoing CV outcomes study, Researching CV Events with a Weekly Incretin in Diabetes (REWIND), will further assess CV safety of dulaglutide.

**Electronic supplementary material:**

The online version of this article (doi:10.1186/s12933-016-0355-z) contains supplementary material, which is available to authorized users.

## Background

Patients with type 2 diabetes (T2D) have a substantially increased lifetime risk for cardiovascular (CV) disease and associated death; older persons with T2D may have twofold to fourfold greater risk than those without diabetes [1–5]. Concerns have been raised regarding the effects of different anti-diabetic medications on CV safety [6], therefore, regulatory agencies now require that the CV safety of anti-diabetic medications be thoroughly studied prior to regulatory review [Food and Drug Administration (FDA) 2008; Committee for Medicinal Products for Human Use (CHMP) 2012] [7, 8]. It is advised to perform a meta-analysis of the clinical development data to demonstrate that the upper bound of the 95 % confidence interval (CI) of the hazard ratio (HR) for major adverse CV events in the treatment group is <1.8 compared to the comparator therapy (control) group. Additionally, it is advised to demonstrate that the upper bound of the 95 % CI of the HR is <1.3 in a dedicated CV outcomes study.

Glucagon-like peptide-1 receptor agonists (GLP-1 RAs) significantly decrease hemoglobin A1c (HbA1c) via stimulating glucose-dependent insulin secretion, suppressing glucagon, delaying gastric emptying, and reducing appetite and food intake [9]. Early long-term glycemic control may decrease macrovascular complications in T2D [10, 11], therefore, treatment with GLP-1 RAs may modify CV risk. In addition to glycemic control, treatment with GLP-1 RAs exerts additional effects that may potentially alter the CV risk, including a decrease in body weight and a small decrease in systolic blood pressure (SBP), but with a small increase in heart rate.

Dulaglutide, a once-weekly long-acting GLP-1 RA, exhibits GLP-1 mediated effects [12, 13]. The Assessment of Weekly AdministRation of dulaglutide in Diabetes (AWARD) clinical development program demonstrated that treatment with dulaglutide improved glycemic control and caused significant decreases in HbA1c when compared to placebo or active comparators [14–23]. Treatment with dulaglutide was also associated with effects that may potentially alter CV risk including weight loss (or attenuation of weight gain) [14–23] and a small decrease in SBP, but with a small increase in heart rate [17]. This pre-specified CV meta-analysis evaluated CV risk in the dulaglutide clinical development program and included prospectively blinded adjudicated CV events from 9 controlled clinical studies, using different comparators and background medications, and encompassing a broad spectrum of the T2D population.

## Methods

### Studies

This CV meta-analysis included adjudicated CV events from a total of 9 clinical trials [14–23]; 4 Phase 2 studies (study durations: 12–26 weeks) and 5 Phase 3 studies (study durations: 52–104 weeks) (Table 1). A total of 3885 patients randomized to dulaglutide and a total of 2125 patients randomized to comparator therapy (placebo or active comparator) are included in this analysis. The dose of dulaglutide in the Phase 2 studies and the dose-finding portion of AWARD-5 ranged from 0.1 to 3 mg and it was 0.75 and 1.5 mg in the Phase 3 studies. While all 4 Phase 2 studies were placebo-controlled, comparator agents in the 5 Phase 3 studies included placebo, sitagliptin, exenatide, insulin glargine, or metformin. After completing the study treatment period, patients in these studies discontinued the assigned therapies and did not continue treatment in safety follow-up periods or in separate protocols.

**Table 1:** Phase 2 and Phase 3 dulaglutide studies included in cardiovascular meta-analysis

Study	Reference	Patients randomized		Dulaglutide doses (mg)	Treatment duration (weeks)	Comparators	Background therapy
		All (N)	Dulaglutide (n)				
*Phase 2 studies*							
Dose titration^a^	[14]	262	196	0.5 → 1.0, 1.0, 1.0 → 2.0	16	Placebo	2 OAMs
Monotherapy^a^	[15]	167	135	0.1, 0.5, 1.0, 1.5	12	Placebo	None
Japanese study^a^	[16]	145	108	0.25, 0.5, 0.75	12	Placebo	None
ABPM study^a^	[17]	755	505	0.75, 1.5	26	Placebo	≥1 OAM
*Phase 3 studies*							
AWARD-1^b^	[18]	978	559	0.75, 1.5	52	Placebo, exenatide	Metformin + pioglitazone
AWARD-2^b^	[19]	810	545	0.75, 1.5	78	Glargine	Metformin + glimepiride
AWARD-3^a^	[20]	807	539	0.75, 1.5	52	Metformin	None
AWARD-4^b^	[21]	884	588	0.75, 1.5	52	Glargine	Lispro ± metformin
AWARD-5^a^	[22, 23]	1202	710	Stage 2: 0.75, 1.5	104	Placebo, sitagliptin	Metformin

*ABPM* ambulatory blood pressure monitoring, *AWARD* Assessment of Weekly AdministrRation of LY2189265 (dulaglutide) in Diabetes, *OAM* oral anti-diabetic medication

^a^Double-blind studies

^b^Open-label studies

As previously indicated in original publications for each clinical trial, all patients provided written informed consent before initiation of study procedures. Institutional review boards of all participating sites approved the protocol. Trials were conducted in compliance with Good Clinical Practice guidelines and the ethical principles stated in the Declaration of Helsinki [14–23].

The Preferred Reporting Items for Systematic Reviews and Meta-Analyses (PRISMA) criteria for reporting meta-analyses [24] were followed; however, this meta-analysis was based on a pre-specified meta-analysis plan specifying that the analysis would include Phase 2 and Phase 3 studies from the dulaglutide clinical development program. Therefore, many of the PRISMA criteria related to reporting literature search strategy and criteria used for inclusion or exclusion of studies did not apply for this pre-specified meta-analysis.

This meta-analysis was performed on patient-level data from the included studies and all CV events were prospectively collected and adjudicated based on a common charter and based on the same event definitions. As prospectively defined, data from dulaglutide clinical pharmacology studies were not included in the meta-analysis due to their shorter durations of exposure (<6 weeks). There were no investigator-reported events of CV-related death, non-fatal stroke, non-fatal MI, or hospitalization for unstable angina in these studies.

### Analysis population

Common inclusion criteria in the Phase 3 trials included a diagnosis of inadequately controlled T2D, age ≥18 years, a baseline HbA1c of 6.5–11.0 % (depending on specific study), previous treatment with diet and exercise alone or ongoing treatment with one or more oral anti-diabetic medications (OAM), stable weight (±5 %) for at least 3 months prior to screening, and a written informed consent. Females of childbearing potential were required to agree to use a reliable method of birth control during the study. Patients with recent CV events were generally excluded [for example, myocardial infarction (MI), stroke, or heart failure within 2–6 months]; Phase 2 studies and AWARD-5 study excluded patients with heart failure; AWARD-2 and AWARD-4 only excluded New York Heart Association (NYHA) class III or class IV heart failure; in addition, NYHA class II heart failure was also excluded in AWARD-1 due to concomitant administration of pioglitazone; and AWARD-3 only excluded NYHA class IV heart failure. The majority of studies excluded patients with serum creatinine ≥1.5 mg/dL (male) or ≥1.4 mg/dL (female), or creatinine clearance <60 mL/min. However, 3 studies included patients with higher serum creatinine levels or a lower estimated glomerular filtration rate (eGFR) if not contraindicated with concomitant medications; therefore, some patients with a eGFR < 60 mL/min/1.73 m^2^ were included in the meta-analysis [14, 17, 21]. Entry criteria for each study have been previously published [14–23]. The analysis population included all randomized patients [intent-to-treat (ITT) population].

### Adjudication

An expert panel, blinded to treatment, [Duke Clinical Research Institute (DCRI), Duke University Medical Center] adjudicated the following CV events: death (CV and non-CV), acute coronary syndromes (MI and hospitalization for unstable angina), cerebrovascular events (stroke and transient ischemic attack), coronary revascularization procedures (coronary artery bypass grafting and percutaneous coronary interventions), and hospitalization for heart failure. CV events were initially identified in the 9 included studies by site investigators or study personnel during study conduct. Clinical sites were responsible for reporting applicable adverse events (AEs) and serious adverse events (SAEs), and completing a CV event-specific electronic case report form (eCRF). In addition, clinical trial databases were queried for specific events based on reported AE terms (i.e., MedDRA Preferred Terms) to identify potentially unreported events. CV events were also reported if potentially unreported events were identified by monitoring personnel or adjudicators. All identified CV events were sent to the DCRI clinical event classification (CEC) committee for adjudication. The CEC committee prospectively adjudicated all identified CV events in a blinded manner throughout the course of the studies based on pre-specified criteria and a pre-defined adjudication process.

### Objectives

The primary objective was to compare, for all randomized patients (ITT population), the time from randomization to first occurrence of the composite endpoint of death due to CV causes, nonfatal MI, nonfatal stroke, or hospitalization for unstable angina [4-component Major Adverse CV Event (MACE)] between patients receiving any dose of dulaglutide and those receiving comparator therapy (placebo or active comparator).

Additional objectives evaluated in this study included:

- Time from randomization to first occurrence of CV events observed with once-weekly dulaglutide versus comparator group for each individual component of the composite primary endpoint.
- Time from randomization to first occurrence of the 3-component MACE endpoint (composite of death due to CV causes, nonfatal MI or stroke).
- Time from randomization to first occurrence of the 6-component MACE endpoint (composite of death due to CV causes, nonfatal MI or stroke, hospitalization for unstable angina or heart failure, or coronary revascularizations).
- Time from randomization to first occurrence of hospitalization for heart failure, coronary revascularizations, and all-cause mortality.

All analyses including the subgroup analysis were conducted on all randomized patients (ITT population). Comparisons of dulaglutide versus placebo or active comparators, and dulaglutide dose (0.75 or 1.5 mg) versus placebo or all comparators, were also conducted. Two sensitivity analyses were conducted for the per-protocol (PP) and completers populations. The PP population was defined as all randomized patients who had not discontinued study drug or discontinued from the study, had an overall compliance of ≥75 %, and had no important protocol deviations. The completers population was defined as all randomized patients who completed a given study regardless of compliance with the protocol.

### Statistical analysis

The primary analysis was a Cox proportional hazards regression model stratified by study (stratum). The model included treatment as a fixed effect with only 2 levels for the factor (dulaglutide or comparator groups). Stratification by study was used to account for potential differences in the HRs due to differing durations of the Phase 2 and 3 trials and differing lengths of follow-up periods. Thus, all Phase 2 studies were combined into one stratum; each of the Phase 3 AWARD studies (Table 1) formed an individual stratum. Since there were no patients with an adjudicated primary CV endpoint event in the comparator therapy arm of AWARD-3, it was combined with AWARD-5 into a single stratum. Statistical heterogeneity between the strata was tested by including in the primary analysis model an interaction term between treatment and strata.

All adjudicated CV events during the treatment periods and up to 30 days after treatment discontinuation were included in the analyses. Unless otherwise noted, all tests of treatment effects were conducted at a 2-sided alpha level of 0.05 and the CI was calculated at a 2-sided 95 % level. The pre‐specified meta‐analysis plan included a second potential meta‐analysis in case the upper bound of the CI for the HR was not <1.8 in the first meta‐analysis; adjustment for multiplicity was performed for the primary endpoint using the Pocock alpha-spending function to control for type 1 error. Tests of treatment effects for the primary endpoint were conducted at a 2-sided alpha level of 0.0198 and the CI was calculated at a 2-sided 98.02 % level. The second meta‐analysis was not performed since this first meta-analysis showed the upper bound of the 98.02 % CI for the HR was <1.8. All tests of interactions between treatment groups and other factors were conducted at a 2-sided alpha level of 0.10.

Counts and proportions of patients who experienced a primary endpoint and person-years of follow-up for the primary endpoint and the incidence rates were calculated. The incidence rate was calculated by dividing the number of patients who developed the event during the study period by the event-specific person-years of follow-up. In addition, Kaplan–Meier plots were reported for the primary endpoint, as well as Forest plots of the results by study (stratum).

Sensitivity analyses consisted of a re-evaluation of the primary endpoint for the PP and completers populations. Subgroup analysis, using the Cox proportional hazards regression model, included the subgroups of sex, age (≤65, >65 years), duration of diabetes (<5, [5–10], >10 years), prior history of CV disease, prior history of hypertension, prior history of hyperlipidemia, body mass index (BMI; <30, ≥30 kg/m^2^), baseline HbA1c [<8 % (<63.9 mmol/mol), ≥8 % (≥63.9 mmol/mol)], race (White, non-White), and geography (North America, South America, Europe, Asia Pacific, other). As a sensitivity analysis, MedDRA preferred terms were used to categorize AE reflecting CV specific events, regardless of their adjudication outcome. All analyses were implemented using SAS^®^ Version 8.2 or higher.

## Results

### Demographic and baseline disease characteristics

This CV meta-analysis included a total of 6010 patients: 3885 treated with dulaglutide and 2125 treated with comparator therapy (placebo or an active) (Table 2). The mean age of patients was 56.1 years and the majority of patients were <65 years of age (81.6 %) and over two-thirds were White (68.3 %); 31.1 % of the patients were Hispanic or Latino. More than half (55.7 %) were enrolled in North America; 21.9 % were enrolled in the European Union (EU), 10.1 % in South America, 6.7 % in Asia Pacific, and 5.6 % in other regions. The overall proportions of males (51.2 %) and females (48.8 %) and mean BMI (32.3 kg/m^2^) were similar in the dulaglutide and comparator groups.

**Table 2:** Demographic and other baseline characteristics of patients included in the cardiovascular meta-analysis—all randomized patients (ITT population)

Variable	All comparators (N = 2125)	All dulaglutide (N = 3885)	Total (N = 6010)	p value^a^
Mean age (years)	56.0	56.2	56.1	0.78
Age group [n (%)]	–	–	–	0.30
<65 years	1724 (81.1)	3177 (81.8)	4901 (81.6)	
≥65 years	401 (18.9)	708 (18.2)	1109 (18.5)	
Sex [n (%)]	–	–	–	0.27
Female	1016 (47.8)	1916 (49.3)	2932 (48.8)	
Male	1109 (52.2)	1969 (50.7)	3078 (51.2)	
Ethnicity [n (%)]	–	–	–	0.23
Hispanic or Latino	660 (31.1)	1209 (31.1)	1869 (31.1)	
Not Hispanic or Latino	1463 (68.9)	2676 (68.9)	4139 (68.9)	
Race^b^ [n (%)]	–	–	–	0.27
American Indian or Alaska Native	135 (6.4)	231 (6.0)	366 (6.1)	–
Asian	271 (12.8)	509 (13.1)	780 (13.0)	–
Black or African American	113 (5.3)	245 (6.3)	358 (6.0)	–
White	1446 (68.1)	2656 (68.4)	4102 (68.3)	–
Mean duration of diabetes (years)	8.0	7.9	7.9	0.22
Duration of diabetes	–	–	–	0.01
<5 years	765 (36.0)	1494 (38.5)	2259 (37.6)	
≥5 and <10 years	659 (31.0)	1088 (28.0)	1747 (29.1)	
≥10 years	701 (33.0)	1303 (33.5)	2004 (33.3)	
Mean BMI (kg/m^2^)	32.4	32.3	32.3	0.51
Fasting plasma glucose				
Mean (mmol/L)	9.07	9.01	9.04	0.69
<5.6 mmol/L [n (%)]	109 (5.2)	200 (5.2)	309 (5.2)	0.79
≥5.6 mmol/L [n (%)]	1994 (94.8)	3630 (94.8)	5624 (94.8)	
HbA1c				
Mean (%)	8.1	8.1	8.1	0.87
Mean (mmol/mol)	65.0	65.0	65.0	
<8 % (<63.9 mmol/mol) [n (%)]	1113 (52.6)	2039 (52.6)	3152 (52.6)	0.62
≥8 % (≥63.9 mmol/mol) [n (%)]	1005 (47.5)	1838 (47.4)	2843 (47.4)	

*BMI* body mass index, *HbA1c* glycosylated hemoglobin A1c

^a^Categorical variables were compared between treatments by Cochran–Mantel–Haenszel test. Continuous variables were compared between treatments by ANOVA model adjusting for strata: variable = treatment + stratum

^b^Categories omitted from the table: Native Hawaiian or Other Pacific Islander (0.1 % total), multiple (0.9 % total), and unknown (5.7 % total)

The 2 treatment groups were similar with respect to most baseline characteristics including mean HbA1c (8.1 %), fasting plasma glucose (FPG; 9.04 mmol/L), and duration of diabetes (7.9 years) (Table 2). There was a small, but significant difference between treatment groups with respect to subgroups defined by baseline duration of diabetes (i.e., <5 years for 38.5 % dulaglutide vs. 36.0 % comparators; ≥5 to <10 years for 28 % dulaglutide vs. 31 % comparators; p = 0.012).

In addition, baseline CV risk factors or prior CV disease including history of smoking, hypertension, hyperlipidemia, prior stroke/transient ischemic attack, and multiple other CV interventions, as well as renal function were similar between the two treatment groups (Table 3). However, prior MI at baseline was slightly higher for the dulaglutide group compared to the comparator group (3.4 vs. 2.4 %, p = 0.049) (Table 3).

**Table 3:** Comparison of cardiovascular risk factors at baseline—all randomized patients (ITT population)

Variable	All comparators (N = 2125) n (%)	All dulaglutide (N = 3885) n (%)	Total (N = 6010) n (%)	p value^a^
Prior MI	51 (2.4)	132 (3.4)	183 (3.0)	0.049
History of unstable angina	34 (1.6)	55 (1.4)	89 (1.5)	0.632
Prior coronary revascularization	65 (3.1)	115 (3.0)	180 (3.0)	0.738
History of stroke or TIA	35 (1.7)	63 (1.6)	98 (1.6)	0.979
History of heart failure	12 (0.6)	16 (0.4)	28 (0.5)	0.284
History of (documented) coronary artery disease	86 (4.1)	189 (4.9)	275 (4.6)	0.163
Has hypertension	1357 (63.9)	2451 (63.1)	3808 (63.4)	0.504
Has hyperlipidemia^b^	1176 (55.3)	2116 (54.5)	3292 (54.8)	0.296
History of carotid revascularization^c^	4 (0.2)	8 (0.2)	12 (0.2)	0.863
History of lower extremity arterial revascularization^c^	7 (0.5)	8 (0.3)	15 (0.3)	0.380
History of peripheral vascular disease^c^	30 (1.4)	57 (1.5)	87 (1.5)	0.962
History of atrial fibrillation^c^	30 (1.4)	38 (1.0)	68 (1.1)	0.173
Current smoker	335 (15.9)	551 (14.3)	886 (14.8)	0.101
Current smoker with hypertension and hyperlipidemia	138 (6.5)	217 (5.6)	355 (5.9)	0.161
Kidney function group by eGFR	–	–	–	0.899
<30 mL/min/1.73 m^2^	1 (0.1)	2 (0.1)	3 (0.1)	
30 ≤ eGFR < 60 mL/min/1.73 m^2^	126 (5.9)	228 (5.9)	354 (5.9)	
≥60 mL/min/1.73 m^2^	1998 (94.0)	3654 (94.1)	5652 (94.1)	
Albuminuria group by urine ACR^d^	–	–	–	0.433
<30 mg/g	1566 (76.8)	2811 (77.1)	4377 (77.0)	
30 ≤ Urine ACR ≤ 300 mg/g	395 (19.4)	713 (19.6)	1108 (19.5)	
>300 mg/g	79 (3.9)	121 (3.3)	200 (3.5)	

*eGFR* estimated glomerular filtration rate, *HDL* high density lipoprotein, *LDL* low density lipoprotein, *MI* myocardial infarction, *TIA* transient ischemic attack, *urine ACR* urinary albumin/creatinine ratio

^a^Treatments were compared by Cochran–Mantel–Haenszel test. Strata = studies

^b^Having hyperlipidemia or taking lipid lowering drugs at baseline or having LDL-C ≥160 mg/dL or HDL-C <40 mg/dL or triglycerides ≥200 mg/dL. Missing value was set to category ‘No’

^c^CV risk factors were either collected directly or were identified by searching historical events and pre-existing events at baseline with relevant preferred terms. Missing value is set to category ‘No’. History of lower extremity arterial revascularization was not collected, could not be defined, and is missing for the dose titration study, the monotherapy study, and AWARD-5 study

^d^Urine ACR was not collected in the dose titration study

### Effect on primary 4-component MACE and individual components

Results of the meta-analysis showed that 26 (0.67 %) patients in the dulaglutide group and 25 (1.18 %) patients in the comparator group experienced at least 1 of the 4 major CV events (death due to CV causes, nonfatal MI, nonfatal stroke, or hospitalization for unstable angina) (Table 4). The estimated HR for the comparison was 0.57 with an adjusted 98.02 % CI of 0.30, 1.10 (p = 0.046), indicating that there was no significant difference between treatment groups. These data suggest that treatment with dulaglutide was not associated with an increase in the risk of experiencing a 4-component MACE endpoint versus comparator therapy. In addition, the upper bound of the adjusted 2-sided 98.02 % CI for the HR (1.10) was less than the FDA-stipulated limit of 1.8. The treatment difference was also consistent across the strata (studies); there was no significant interaction between treatment and strata (interaction p = 0.598).

**Table 4:** Time-to-event analysis of the primary cardiovascular (CV) endpoint and individual components—all randomized patients (ITT population)

Endpoint component	All comparators (N = 2125) n (%)	All dulaglutide (N = 3885) n (%)	HR^a^ (adj. 98.02 % CI)	p value^a^
Primary 4-component MACE endpoint	25 (1.18)	26 (0.67)	0.57 (0.30, 1.10)	0.046
Death from CV causes^b^	5 (0.24)	3 (0.08)	0.35 (0.07, 1.87)	0.119
Nonfatal MI	14 (0.66)	9 (0.23)	0.35 (0.13, 0.95)	0.014
Nonfatal stroke	4 (0.19)	12 (0.31)	1.61 (0.42, 6.20)	0.411
Hospitalization for unstable angina	6 (0.28)	3 (0.08)	0.28 (0.05, 1.46)	0.054
Exposure (event specific person-years follow-up)	2211.31	3926.90	–	–
Incidence rate per 100 person-years	1.13	0.66	–	–

*AWARD* Assessment of Weekly AdministrRation of LY2189265 (dulaglutide) in Diabetes, *CV* cardioavascular, *HR* hazard ratio, *MACE* major adverse CV event, *MI* myocardial infarction

^a^Calculated from a stratified Cox Proportional Hazards regression model: response = treatment. Strata = studies; all Phase 2 studies formed one stratum, AWARD-3 and AWARD-5 formed one stratum. 2-sided p value to be compared to an alpha level of 0.0198 for test of superiority

^b^Death from CV causes is defined as a death resulting from an acute MI, sudden cardiac death, death due to heart failure, death due to stroke, and death due to other CV causes

**Fig. 1:**
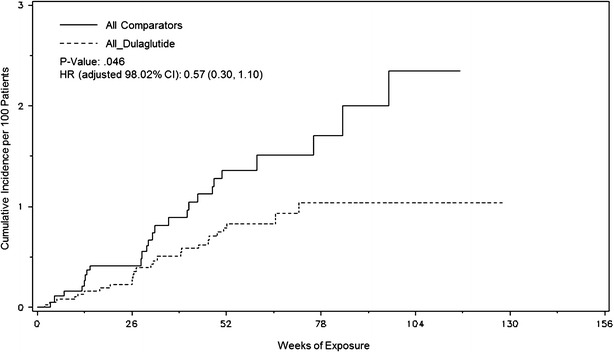
Time to first primary 4-component MACE. A Kaplan–Meier plot (with estimated HR [98.02 % CI] and *p* value) illustrating the time in weeks from randomization to the first occurrence of any of the 4 components of the primary endpoint measure

**Fig. 2:**
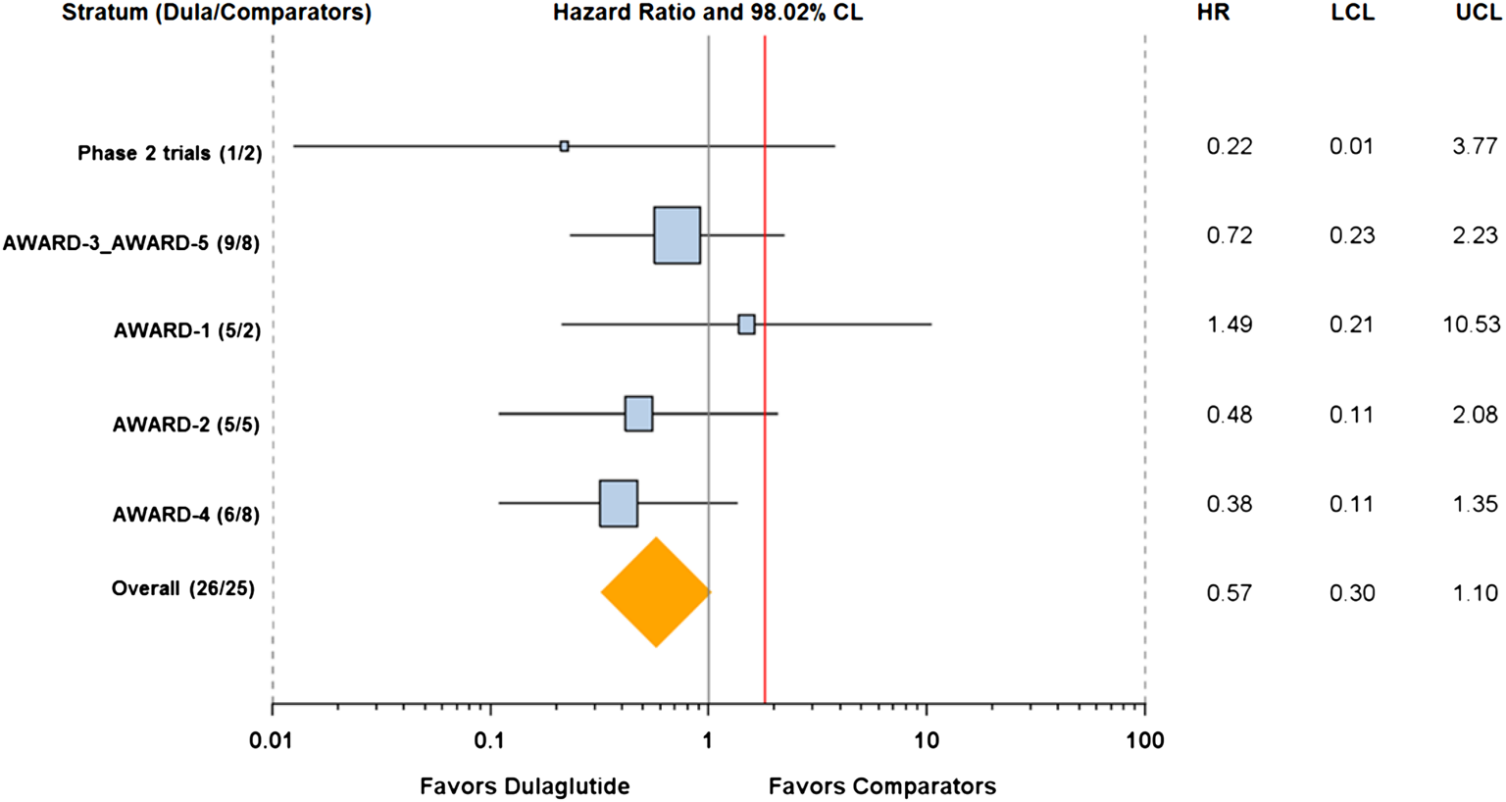
Forest plot of the primary 4-component MACE endpoint by stratum. A comparison of the primary analysis results (HR [98.02 % CI]) in each stratum (study or combinations of studies by which the primary analysis was stratified) with the overall result. *Numbers* of CV events per each treatment group (Dula/Comparators) are indicated in the parentheses in the y-axis under Stratum

The Kaplan–Meier curves for the estimated cumulative incidence of the time to first occurrence of the 4-component MACE appear to start separating early, with the earliest divergence appearing around 28 weeks of exposure to treatment (Fig. 1). A Forest plot of the primary 4-component MACE comparing HR values with 98.02 % CIs by stratum (study) shows that the HRs for dulaglutide compared to comparator therapy are generally <1.0 which is consistent with the overall result (Fig. 2).

There was no significant difference between the two treatment groups for the risk of death due to CV causes, nonfatal stroke, or hospitalization for unstable angina. However, the relative risk of experiencing a nonfatal MI was significantly lower in the dulaglutide group compared with the comparator group (estimated HR 0.35; adjusted 98.02 % CI [0.13, 0.95; p = 0.014]) (Table 4).

### Results of the sensitivity analyses for the primary 4-component MACE endpoint

Time-to-event sensitivity analysis results for the PP and completers populations (Additional file 1: Tables S1 and S2, respectively) showed no significant differences between the 2 treatment groups with respect to the composite 4-component MACE endpoint. A total of 24 primary 4-component MACE events were reported in the PP population; 13 occurred in the dulaglutide group and 11 in the comparator group, with no significant difference between the 2 groups (HR 0.63; 98.02 % CI 0.24, 1.63; p = 0.26). Results in the completers population were consistent with the PP analysis.

Consistent with the primary comparison, there was no significant difference in the time to occurrence of the 4-component MACE by dulaglutide (all doses) versus active comparators or dulaglutide (all doses) versus placebo (Additional file 1: Table S3). Furthermore, no significant differences were observed when comparing dulaglutide dose (1.5 or 0.75 mg) versus placebo or dulaglutide dose versus all comparator therapy (active and placebo) (Additional file 1: Table S3).

### Results of additional endpoints analyses

No significant differences were observed between the two treatment groups for the risk of experiencing all-cause mortality (HR 0.50; 95 % CI 0.18, 1.38), composite 3-component MACE, or heart failure requiring hospitalization (Table 5). However, there was a significant difference between the treatment groups for the 6-component MACE endpoint (HR 0.57; 95 % CI 0.37, 0.90; p = 0.016) and the time to coronary revascularization endpoint (HR 0.44; 95 % CI 0.21, 0.92; p = 0.029). No other significant differences were observed between the 2 treatment groups.

**Table 5:** Time-to-event analysis of other cardiovascular (CV) endpoints—all randomized patients (ITT population)

Endpoint component	All comparators (N = 2125) n (%)	All dulaglutide (N = 3885) n (%)	HR^a^ Est. (95 % CI)	p value^a^
All cause mortality	8 (0.38)	7 (0.18)	0.50 (0.18, 1.38)	0.181
3-Component MACE endpoint^b^	21 (0.99)	23 (0.59)	0.60 (0.33, 1.08)	0.090
6-Component MACE endpoint^c^	37 (1.74)	39 (1.00)	0.57 (0.37, 0.90)	0.016
Heart failure requiring hospitalization	2 (0.09)	7 (0.18)	2.02 (0.41, 9.88)	0.378
Coronary revascularization	16 (0.75)	13 (0.33)	0.44 (0.21, 0.92)	0.029
Percutaneous coronary intervention	14 (0.66)	11 (0.28)	0.43 (0.19, 0.95)	0.036
Coronary artery bypass grafting	2 (0.09)	2 (0.05)		

*AWARD* Assessment of Weekly AdministrRation of LY2189265 (dulaglutide) in Diabetes, *CV* cardioavascular, *HR* *Est* estimated hazard ratio, *MACE* major adverse CV event, *MI* myocardial infarction

^a^Calculated from a stratified Cox Proportional Hazards regression model: response = treatment. Strata = studies. All Phase 2 studies form one stratum, AWARD-3 and AWARD-5 form one stratum. When the total number of outcomes is <10, survival analysis is not performed. Instead when the total number of outcomes is <10 and ≥5, Mantel–Haenszel odds ratio and p value by Cochran–Mantel–Haenszel test are reported; When the total number of outcomes is <5, ratio and p-value are not reported

^b^Composite endpoint of death from CV causes, nonfatal MI, or nonfatal stroke

^c^Composite endpoint of death from CV causes, nonfatal MI, nonfatal stroke, hospitalization for unstable angina, coronary revascularization, or heart failure requiring hospitalization

### Subgroup analysis

The results of the subgroup analysis for the ITT population indicated that there was no treatment by subgroup interaction and use of dulaglutide resulted in a consistent effect across subgroups (data not shown). The HR point estimate was <1.0 for all subgroups tested (favoring dulaglutide) except for tobacco use (HR 1.38), however, this effect was not significant (95 % CI 0.41, 4.58).

### Overall CV adverse events post-baseline (regardless of adjudication)

As a sensitivity analysis, AE preferred terms were used to identify certain categories of CV AEs including coronary artery disease, angina, atrial fibrillation, MI, stroke or transient ischemic attack, heart failure, coronary revascularization, and carotid revascularization. Proportions of patients with ≥1 CV AE, reported post-baseline regardless of adjudication, were comparable between the dulaglutide (8.6 %) and the comparators (9.1 %) groups. No significant differences were observed in any category between the two groups (data not shown).

## Discussion

In consideration of the substantial risk for CV events in persons with diabetes, regulatory agencies have required rigorous assessment of CV safety for new diabetic medications [7, 8]. Although, data from final CV outcome studies are still limited for the GLP-1 class, retrospective and pre-specified CV meta-analyses support continued clinical use of this class while awaiting confirmatory CV outcome data. Currently, only one GLP-1 RA (lixisenatide) has reported CV outcome study (ELIXA) results. This study showed that treatment of patients with a prior CV event with lixisenatide does not alter the risk for CV events [25].

### CV meta-analysis results for dulaglutide and other GLP-1RAs

This CV meta-analysis included data from 9 controlled dulaglutide clinical studies with different comparators, background medications, and covering a broad spectrum of the T2D population. The baseline demographics and CV risk characteristics were comparable between the dulaglutide and comparator group. However, prior MI at baseline was slightly higher for the dulaglutide group compared to the comparator group (3.4 vs. 2.4 %, p = 0.049), an effect that would not be expected to introduce favorable bias for dulaglutide.

Overall, this CV meta-analysis suggests that treatment with dulaglutide was not associated with an increase in the risk of experiencing a MACE endpoint compared with comparator therapies. The incidence of the 4-component MACE in the dulaglutide group over time was consistently lower than the comparator group (Fig. 1). The results of the dulaglutide CV meta-analysis are consistent with other GLP-1 RAs CV meta-analyses data. Retrospective post hoc CV meta-analyses demonstrated that treatment with shorter-acting GLP-1 RAs (liraglutide or exenatide) did not increase the risk of MACE in patients with T2D [26, 27]. Recent pre-specified CV meta-analyses of prospectively adjudicated events also suggest that treatment with the longer-acting GLP-1 RAs, albiglutide and taspoglutide, does not increase the risk of MACE in patients with T2D [28, 29].

Evaluation of individual component endpoints demonstrated no significant difference between dulaglutide and comparators for risk of death from CV causes, non-fatal stroke, or hospitalization for unstable angina. The incidence for non-fatal stroke was numerically higher in the dulaglutide group compared to the comparator group, but with a HR 98.02 % CI that ranged from 0.42 to 6.20. On the other hand, the relative risk of experiencing a nonfatal MI was significantly lower with the dulaglutide group compared with the comparator group (estimated HR: 0.35; adjusted 98.02 % CI [0.13, 0.95; p = 0.014]). However, the total number of events was small and therefore, the interpretation of these results should be done with caution.

Additional endpoints including the 3-component MACE and the 6-component MACE also show results consistent with results of the primary measure, indicating that treatment with dulaglutide does not increase the risk for MACE whether defined narrowly (3-component MACE) or broadly (6-component MACE). Evaluation of individual component endpoints showed a significant decrease in the risk for the combined coronary revascularization endpoint (HR 0.44; 95 % CI [0.21, 0.92]). No significant difference was observed for the heart failure requiring hospitalization endpoint wherein the estimated HR was 2.02 and the 95 % CI was [0.41, 9.88]. Furthermore, additional analyses conducted to examine the primary 4-component MACE endpoint in the PP and completers population, different dulaglutide doses (0.75 and 1.5 mg), and subgroup analyses are all consistent with the outcome of the primary analysis observed in all randomized patients.

The incidence for major CV events (3-component, 4-component, and 6-component MACE) ranged from 0.59 to 1.00 % in the dulaglutide group compared to 0.99–1.74 % in the comparator group. This incidence rate of MACE events is consistent with rates observed in recent CV meta-analyses, which evaluated liraglutide, albiglutide, taspoglutide, saxagliptin, and vildagliptin [26, 28–31]. Incidence rates per 100 person-years for all major CV endpoints (the primary 4-component MACE) were lower in the dulaglutide group compared with the comparators group (0.66 vs. 1.13, respectively).

### Cardiovascular effects of GLP-1 RAs

Pre-clinical and recent human data indicate that GLP-1 RAs may play a cardioprotective role via reduction in myocardium infarct size, improving endothelial function, and vasodilation [32–35]. However, another study showed that chronic treatment with liraglutide for 14 weeks did not affect endothelial function [36] indicating that this may be limited to acute effect. Recent clinical data indicate that GLP-1 RAs may alter other modifiable CV risk factors such as body weight, blood pressure, or lipids [32, 37] potentially leading to reduction in macrovascular risk [38]. It is unknown if these effects would prevent CV events, this hypothesis remains to be confirmed in long-term CV outcomes studies.

### Potential alteration of CV risk factors by dulaglutide or other anti-hyperglycemic treatments, effects of long-term glycemic control, and the need for long-term data

In all of the 9 Phase 2 and Phase 3 studies included in this meta-analysis, treatment with dulaglutide improved glycemic control compared to placebo or active comparators [14–23]. Consistent with the GLP-1 RAs’ mechanism of action, treatment with dulaglutide also has been associated with alterations of other additional parameters that may be implicated in modifying CV risk including lower risk of hypoglycemia [39], body weight loss [39], and a small decrease in SBP [17]. Although T2D is an established risk factor for macrovascular disease, several studies demonstrated that intensive glycemic control only resulted in an insignificant slight decrease in CV endpoints [40, 41]. However, a recent placebo-controlled study in patients with T2D at high risk for CV events demonstrated that a lower rate of 3-component MACE was observed when empagliflozin was added to standard care compared to placebo [42]. Although rates of MI and stroke were similar between the two treatment groups, empagliflozin-treated patients had significantly lower rates of CV-related mortality (3.7 vs. 5.9 % in the placebo group; 38 % relative risk reduction), hospitalization for heart failure (2.7 and 4.1 %, respectively; 35 % relative risk reduction), and all-cause mortality (5.7 and 8.3 %, respectively; 32 % relative risk reduction). In addition to glycemic efficacy, treatment with empagliflozin has been also associated with body weight loss, diuretic effect, and a decrease in blood pressure [42], effects that may lead to an overall lowering of CV risk. Furthermore, long-term follow-up data after intensive glycemic control in patients at early stage of T2D showed significant decrease in MI and all-cause mortality [10], indicating that early glycemic control may play a significant role in modulating long-term CV outcomes and delaying diabetes-related macrovascular complications. The recent 10-year long-term follow-up from the Veteran Affairs Diabetes Trial (VADT) study provides additional evidence that the CV benefits from the initial intensive glucose control (median of 5.6 years) is also observed for patients with a long duration of T2D (mean of 11.6–12 years) [43]. After nearly 10 years of follow-up, patients in the VADT trial who were randomized to 5.6 years of intensive glucose control had a significantly lower risk (17 % reduction; p = 0.04) of having a major CV event than those given standard therapy. Therefore, early treatment and long-term follow up data may be helpful in the evaluation of the potential CV benefit related to the glycemic effects of GLP-1 RAs.

### Strengths and limitations of this CV meta-analysis

Among strengths of this CV meta-analysis is the inclusion of a broad population that is representative of the general T2D population. This meta-analysis included patients at different stages of T2D, with a mean duration of diabetes ranging from 2.6 to 12.7 years and with multiple CV risk factors [14–23]. Although this CV meta-analysis suggests that dulaglutide treatment is not associated with excess CV risk, limitations of CV meta-analyses in general include the relatively short treatment duration. In addition, inclusion of a diverse population at different stages of T2D, while appropriate for evaluation of CV safety in patients with different CV risk factors, may not enable evaluation of potential CV benefits due to the limited number of patients at earlier stages of T2D and the relatively short-term follow up. It is also important to note that patients with a recent history of clinically significant and potentially unstable CV disease were excluded from the dulaglutide clinical trial program. For example, patients with congestive heart failure (mainly NYHA class III or IV), or certain recent CV events including MI or stroke, were excluded from most studies. In addition, patients with eGFR < 60 mL/min/1.73 m^2^ were also excluded from most studies. Therefore, certain populations with high CV risk may have been excluded from the overall population included in this CV meta-analysis, which is consistent with most other development programs [26–29]. Accordingly, the low rate of CV events observed in the Phase 2 and 3 T2D populations is a common limitation for these CV meta-analyses in general.

## Conclusions

This CV meta-analysis suggests that treatment of patients with T2D with once-weekly dulaglutide for up to 104 weeks does not increase the risk for CV events. In addition, all sensitivity analyses and additional endpoints consistently suggest that dulaglutide does not increase the risk for CV events. Definitive data regarding the assessment of effects of long-term treatment with dulaglutide on CV risk will be provided by the currently ongoing, large CV outcome Researching CV Events with a Weekly Incretin in Diabetes (REWIND) study.

## Additional file

10.1186/s12933-016-0355-z Supplementary tables.

## Abbreviations

AE: adverse events
AWARD: Assessment of Weekly AdministRation of LY2189265 (dulaglutide) in Diabetes
BMI: body mass index
CEC: clinical event classification
CHMP: Committee for Medicinal Products for Human Use
CI: confidence interval
CV: cardiovascular
DCRI: Duke Clinical Research Institute
eCRF: electronic case report form
eGFR: estimated glomerular filtration rate
EU: European Union
FDA: Food and Drug Administration
FPG: fasting plasma glucose
GLP-1 RA: glucagon-like peptide-1 receptor agonist
HbA1c: hemoglobin A1c
HDL: high-density lipoprotein
HR: hazard ratio
ITT: intent-to-treat
MACE: major adverse cardiovascular event
MI: myocardial infarction
NYHA: New York Heart Association
OAM: oral anti-diabetic medications
PP: per protocol
PRISMA: Preferred Reporting Items for Systematic Reviews and Meta-Analyses
REWIND: Researching Cardiovascular Events with a Weekly Incretin in Diabetes
SAE: serious adverse events
SBP: systolic blood pressure
T2D: type 2 diabetes
VADT: Veteran Affairs Diabetes Trial
