# Supplementary material for: Cardiovascular safety for once-weekly dulaglutide in type 2 diabetes: a pre-specified meta-analysis of prospectively adjudicated cardiovascular events
Source: Cardiovasc Diabetol. 2016 Feb 24;15:38. doi: 10.1186/s12933-016-0355-z (PMC4765050; doi:10.1186/s12933-016-0355-z)
Supplement: Supplementary file 1 — 10.1186/s12933-016-0355-z Supplementary tables. [file 12933_2016_355_MOESM1_ESM.docx]

**Supplementary Tables**

**Table S1 Time-to-event analysis of primary cardiovascular (CV) endpoint and individual components – Per protocol population^a^**

| **Endpoint**  **Component** | **All Comparators  (N = 1353) n (%)** | **All Dulaglutide  (N = 2628) n (%)** | **HR^b^ Est. (adj. 98.02% CI)** | **p-value^b^** |
| --- | --- | --- | --- | --- |
| **Primary 4-Component MACE Endpoint** | **11 (0.81)** | **13 (0.49)** | **0.63 (0.24, 1.63)** | **0.255** |
| Death from CV Causes^c^ | 0 (0.00) | 0 (0.00) | - | - |
| Nonfatal MI | 6 (0.44) | 6 (0.23) | 0.55 (0.14, 2.13) | 0.308 |
| Nonfatal Stroke | 2 (0.15) | 6 (0.23) | 1.45 (0.22, 9.57) | 0.639 |
| Hospitalization for Unstable Angina | 4 (0.30) | 1 (0.04) | 0.13 (0.01, 1.81) | 0.035 |

^a^ Per protocol population was defined as all randomized patients who have not discontinued study drug or discontinued from the study, have an overall adherence of ≥75%, and have no important protocol deviations.

^b^ Calculated from a stratified Cox Proportional Hazards regression model: response = treatment. Strata = studies; all phase 2 studies formed one stratum, AWARD-3 and AWARD-5 formed one stratum. 2-sided p-value to be compared to an alpha level of 0.0198 for test of superiority.

^c^ Death from CV causes is defined as a death resulting from an acute MI, sudden cardiac death, death due to heart failure, death due to stroke, and death due to other CV causes.

Abbreviations: AWARD=**A**ssessment of **W**eekly **A**dminist**R**ation of LY2189265 (dulaglutide) in **D**iabetes; CV=cardioavascular; HR Est=estimated hazard ratio; MACE=major adverse CV event; MI=myocardial infarction.

**Table S2 Time-to-event analysis of primary cardiovascular (CV) endpoint and individual components – Completers population^a^**

| **Endpoint**  **Component** | **All Comparators  (N = 1662) n (%)** | **All Dulaglutide  (N = 3094) n (%)** | **HR^b^ Est. (adj. 98.02% CI)** | **p-value^b^** |
| --- | --- | --- | --- | --- |
| **Primary 4-Component MACE Endpoint** | **15 (0.90)** | **15 (0.48)** | **0.54 (0.23, 1.26)** | **0.089** |
| Death from CV Causes^c^ | 0 (0.00) | 0 (0.00) |  |  |
| Nonfatal MI | 9 (0.54) | 7 (0.23) | 0.43 (0.13, 1.38) | 0.091 |
| Nonfatal Stroke | 3 (0.18) | 7 (0.23) | 1.22 (0.24, 6.09) | 0.777 |
| Hospitalization for Unstable Angina | 5 (0.30) | 1 (0.03) | 0.10 (0.01, 1.38) | 0.013 |

^a^ The “completers” population was defined as all randomized patients who completed a given study regardless of compliance with the protocol. This included patients who did or did not remain on study drug throughout the study. Patients could have discontinued study drug for a variety of protocol-specific reasons (eg, an AE or abnormal laboratory value), but remained in the study. Patients could also have continued on study drug for all study visits, but were noncompliant with other protocol-specific criteria.

^b^ Calculated from a stratified Cox Proportional Hazards regression model: response = treatment. Strata = studies; all phase 2 studies formed one stratum, AWARD-3 and AWARD-5 formed one stratum. 2-sided p-value to be compared to an alpha level of 0.0198 for test of superiority.

^c^ Death from CV causes is defined as a death resulting from an acute MI, sudden cardiac death, death due to heart failure, death due to stroke, and death due to other CV causes.

Abbreviations: AE=adverse event; AWARD=**A**ssessment of **W**eekly **A**dminist**R**ation of LY2189265 (dulaglutide) in **D**iabetes; CV=cardioavascular; HR Est=estimated hazard ratio; MACE=major adverse CV event; MI=myocardial infarction.

**Table S3 Time-to-event analysis of primary 4-component MACE endpoint– Dulaglutide vs. placebo or active comparators/ dulaglutide 0.75 mg and 1.5 mg doses vs. placebo or All Comparators**

| **Comparison** | **N** | **Incidence of**  4-**component** **MACE n (%)** | **HR**  **(98.02% CI)** | **p-value ^a^** |
| --- | --- | --- | --- | --- |
| All Dulaglutide | 3885 | 26 (0.67) | 0.65 (0.32, 1.32) | 0.154 |
| All Active Comparators | 1422 | 19 (1.34) |  |  |
| All Dulaglutide | 3885 | 26 (0.67) | 0.47 (0.09, 2.57) | 0.300 |
| Placebo | 703 | 3 (0.43) |  |  |
| Dulaglutide 1.5 mg | 1700 | 13 (0.76) | 0.59 (0.26, 1.30) | 0.119 |
| All Comparators (active + placebo) | 2125 | 25 (1.18) |  |  |
| Dulaglutide 0.75 mg | 1706 | 13 (0.76) | 0.57 (0.26, 1.27) | 0.104 |
| All Comparators (active + placebo) | 2125 | 25 (1.18) |  |  |
| Dulaglutide 1.5 mg | 1700 | 13 (0.76) | 0.68 (0.10, 4.42) | 0.629 |
| Placebo | 703 | 3 (0.43) |  |  |
| Dulaglutide 0.75 mg | 1706 | 13 (0.76) | 0.47 (0.05, 4.12) | 0.415 |
| Placebo | 703 | 3 (0.43) |  |  |

^a^ Calculated from a stratified Cox Proportional Hazards regression model: response = treatment. Strata = studies; all phase 2 studies formed one stratum, AWARD-3 and AWARD-5 formed one stratum. 2-sided p-value to be compared to an alpha level of 0.0198 for test of superiority.

Abbreviations: AWARD=**A**ssessment of **W**eekly **A**dminist**R**ation of LY2189265 (dulaglutide) in **D**iabetes; CV=cardioavascular; HR Est=estimated hazard ratio; MACE=major adverse CV event; MI=myocardial infarction.
